# Outcome of Heart Failure with Preserved Ejection Fraction: A Multicentre Spanish Registry

**DOI:** 10.2174/157340309789317814

**Published:** 2009-11

**Authors:** Juan C Castillo, Manuel P Anguita1, Manuel Jiménez, on behalf of the BADAPIC group

**Affiliations:** 1 Cardiology Department, Hospital Reina Sofia, Córdoba, Spain; 2 Cardiology Department, Hospital Clínico Virgen de la Victoria, Málaga, Spain

**Keywords:** Heart failure, preserved systolic function, multicentre study.

## Abstract

**Background:**

Studies on clinical features, treatment and prognosis of patients with congestive heart failure (CHF) and preserved left ventricular ejection fraction (LVEF) are few and their results frequently conflicting.

**Aims:**

To investigate the characteristics and long term prognosis of patients with CHF and preserved (≥ 45%) LVEF.

**Methods and Results:**

We conducted a prospective multicentre study with 4720 patients attended in 62 heart failure clinics from 1999 to 2003 in Spain (BADAPIC registry). LVEF was preserved in 30% patients. Age, female gender, prevalence of atrial fibrillation, hypertension and non-ischaemic cardiopathy were all significantly greater in patients with preserved LVEF. Mean follow-up was 40±12 months. Mortality and other cardiovascular complication rates during follow up were similar in both groups. On multivariate analysis ejection fraction was not an independent predictor for mortality. Survival at one and five years was similar in both groups (79% and 59% for patients with preserved LVEF and 78% and 57% for those with reduced LVEF, respectively).

**Conclusions:**

In the BADAPIC registry, a high percentage of heart failure patients had preserved LVEF. Although clinical differences were seen between groups, morbidity and mortality were similar in both groups.

## INTRODUCTION

Congestive heart failure (CHF) is a major health problem and is associated with high morbidity and mortality [1]. Its incidence and prevalence continue to rise due to the gradual ageing of the population, health care improvements and survival of patients with chronic diseases such as hypertension and ischaemic heart disease [2, 3]. It is noteworthy, however, that mortality of patients with CHF has not been significantly reduced despite advances in treatment derived from clinical trials [4, 5]. This is probably due to the greater age of patients and the comorbidity they commonly experience [6]. 

It is estimated that between 20-50% of patients with CHF have preserved systolic function (usually defined as a normal left ventricular ejection fraction [LVEF]) [6-11]. Several studies have reported that in this group there are greater proportions of women, elderly and hypertensive patients than among patients with CHF and reduced LVEF and a smaller proportion of patients with prior myocardial infarction [7, 10, 12].

Controversy exists over whether such patients have better survival than those with CHF and reduced LVEF. Some authors [7, 10, 11, 14] report greater morbidity and mortality among the latter, whereas others [3, 13, 15] report no such findings. The results of previous studies have been inconsistent or conflicting, and the estimates of rates of mortality and readmissions vary widely, since they are derived from heterogeneous populations with different inclusion criteria. 

Eight years ago the Working Group on Heart Failure, Heart Transplantation and Other Therapeutic Alternatives of the Spanish Society of Cardiology set up a voluntary registry of heart failure units, the BADAPIC Registry (an acronym in Spanish for “database of patients with heart failure”). The initial results of the registry have been published elsewhere [16]. In the present study we describe the clinical characteristic and long term survival of patients included in the BADAPIC Registry with preserved LVEF and compared to those with reduced LVEF. 

## METHODS

The BADAPIC Registry is the official registry of the Working Group on Heart Failure, Heart Transplantation and Other Therapeutic Alternatives of the Spanish Society of Cardiology. The registry, set up in 1999 by the Working Group on Heart Failure, is voluntary and so far includes the participation of 62 centres from all over Spain that have specific heart failure units or clinics. The organization and structure of these units vary greatly. The database includes more than 100 variables dealing with the main demographic, clinical and analytical characteristics, as well as functional tests, pharmacological and non-pharmacological therapy, and the evolution of the patient. Since the start of the registry, the data have been collected annually at the end of the year. The data presented here correspond to those collected from 1999 until 2003 and includes the data on 4720 patients from 62 heart failure units or clinics throughout Spain. Among these 4720 patients, 1416 (30%) have preserved ejection fraction, consider as LVEF ≥ 45%. Diagnosis of chronic heart failure was based on the Framingham criteria. For patients with LVEF ≥ 45%, evidence of diastolic impairment assessed by doppler echocardiography was required. 

### Characteristics of the Participating Units

Of the 62 hospitals, 14 (22%) are community hospitals and 48 (78%) general hospitals; 21% of the registered patients were from the community hospitals and 79% from the general hospitals. Only 10 (16%) of the participating hospitals have a heart transplantation program. Although most units are integrated in cardiology departments, eight (13% of total) are managed by internists. 

### Data Collected

For all patients in the study, data were collected during the first visit to the heart failure clinic (either during hospital admission or as an outpatient). Follow-up data was obtained in the following visits to the unit. In the case of patients admitted more than once during the study period, the index admission considered was the first during which systolic function had been evaluated. The frequency of the visits depended on the clinical judgement of each participating physician, although the follow-up data (change in treatment and clinical events) were provided annually by each centre. The follow-up data analysed included mortality, admissions for heart failure, acute myocardial infarction, coronary revascularization, valve surgery or heart transplant. 

### Aetiological Evaluation

The aetiology of heart failure was established in each case by the treating physician at each centre. Ischaemic heart disease was defined by the presence of clinical, electrocardiographic, or angiographic data suggestive of ischaemia or myocardial necrosis. Valve disease was diagnosed if it had been previously diagnosed or if it was indicated by echocardiographic or catheterisation studies (note, however, that the study did not include patients who had been admitted to the unit because of severe valve pathology). Arterial hypertension was diagnosed if it had been previously diagnosed or if the patient had been taking or needed antihypertensive drugs to control blood pressure. Dilated cardiomyopathy was diagnosed if the patient had deteriorated systolic function and a dilated left ventricle but no evidence of ischaemic cardiopathy, valve disease, or arterial hypertension. Although more than one aetiological cause may have been present in a single patient, the physician selected the cause considered to be the most important in that particular patient. Heart failure with preserved systolic function was diagnosed when LVEF was equal to or higher than 45% and reduced systolic function when LVEF was < 45% (whatever the aetiology) measured by echocardiography, radionuclide ventriculography or angiography. Assesment of LVEF was done by echocardiography in 69%, radionuclide ventriculography in 16% and angiography in 15%.

### Statistical Analysis

Qualitative variables are shown as percentages and quantitative variables as means±1 standard deviation. Comparison of the differences between the various subgroups of patients was made using the Chi-square test or Fisher exact test for qualitative variables and the Student t test for continuous variables (all of which showed a normal distribution). The probability of survival and events during the follow-up were estimated by the Kaplan-Meier test and compared using the Mantel log-rank test. Given the different follow-up times of the two subgroups, the incidence of events was adjusted for the total observation time of each; results are expressed as incidence per 100 patients per year of observation. The incidence of events in both groups was compared by the difference in their rates using the Ulm method [17]; the 95% confidence intervals (CI) for these rate differences were determined by the Sahai and Kurshid method [18]. A multivariate analysis using the Cox proportional-hazards method was made. Candidate variables were included in the initial Cox regression model if they were associated with death in an univariate analysis (p<0.1). A p<0.05 was considered statistically significant. 

### Results

As mentioned, the registry included data for 4720 patients enrolled between 1999 and 2003 from 62 heart failure units or clinics throughout Spain. The mean age of patients was 66±12 years. The most common age group was the decade from 70-79 years, with 9% (425 patients) aged 80 years or older; the percentage of patients younger than 50 years of age was very low (11%, 519 patients). Sixty-seven percent of patients (3162) were men and 33% (1558) were women.

Table **1** shows the clinical characteristics of patients in the registry. The most common underlying heart condition among patients with preserved LVEF was arterial hypertension (66%) whereas in the group with reduced LVEF was ischaemic cardiomyopathy (47%). Patients with preserved LVEF were on average 7 years older, were more likely to be female and were more frequently in permanent atrial fibrillation. Hyperlipidemia, coronary artery disease, prior myocardial infarction or revascularization were all more prevalent in the group with reduced LVEF. On the other hand, anemia (haemoglobin < 12 g/dl) was more frequent in patients with preserved LVEF (23% *vs*. 17%, p<0.05) although differences in renal dysfunction was not significantly different between groups (9% *vs*. 10% respectively). 

There were also significant differences between the two groups regarding the medication prescribed after the initial visit to the heart failure unit. As shown in Table **2**, ACE inhibitors, beta blockers and spironolactone were prescribed significantly more often to patients with reduced LVEF. Carvedilol was the most commonly prescribed drug among the beta-blockers, enalapril among the ACE inhibitors, losartan among the ARB type II, furosemide among the diuretics, aspirin among the anti-platelet aggregating agents, amiodarone among the anti-arrhythmic agents, and amlodipine among the calcium antagonists. Only very slight variation was seen during the follow-up in the percentages of the pharmacological agents used. Patients with preserved LVEF received on average less doses for carvedilol (19mg/day on average *vs*. 35 mg/day, p<0.001) and less doses of spironolactone (32 mg/day *vs*. 44mg/day, p<0.001). No significant differences were seen in the dose of enalapril or furosemide. 

### Morbidity and Mortality during Follow-up

Survival data were available for all 4720 patients enrolled in the registry. After a mean follow-up period of 40±12 months, 1880 patients had al least one cardiovascular event. Mean follow-up period was similar between groups (42±13 months in patients with preserved LVEF and 39±12 months in patients with reduced LVEF). A total of 1416 deaths (30%) occurred during follow-up: 912 (64%) patients died because of worsening heart failure, 340 patients (24%) because of sudden death, 99 patients (7%) due to acute myocardial infarction and 65 patients (5%) because of other non cardiovascular reasons. 

Table **3** shows the results of the event incidence analysis for both groups (expressed as numbers per 100 persons per year of observation). Total mortality and mortality due to worsening heart failure were similar between groups as was the incidence of readmission due to cardiovascular problems. Moreover, the incidence of any event was not different in patients with reduced and preserved LVEF (12.1% and 11.7%, respectively). 

The overall survival rate was similar among patients with reduced  LVEF  and  patients with preserved LVEF (Fig (**1**)). The respective mortality rates were 79% and 78% at one year and 59% and 57% at five years. The admission and event-free survival likelihood was similar in both subgroups of patients (see Figs. (**2** and **3**)).

Table **4** lists the results of univariate analyses carried out to determine the effects of several variables on survival. Age, hyperlipemia, anemia, diabetes, hypertension, NYHA class III-IV, atrial fibrillation, ejection fraction, renal dysfunction, no use of beta blockers or statins were all associated with poorer prognosis. Aetiology also affected survival (p<0.001): ischaemic aetiology was related with lower survival time. Multivariate analysis showed survival to be significantly influenced by age, ischaemic aetiology, anemia, renal dysfunction, beta blockers and statins (Table **5**). 

## DISCUSSION

This registry provides a true overall picture of the characteristics and management of heart failure in Spain, within a very well-defined context: patients managed and followed-up in specific heart failure units or clinics. Herein, however, lies its main limitation, as the results can not be extrapolated to the general population. Studies of the differences in clinical characteristics, prognosis and the factors determining prognosis between patients with CHF with preserved and those with reduced LVEF have not all reached the same conclusions, probably related to differences in the design of the study, the methods used to evaluate cardiac function, the duration of follow-up, and in particular the target population (hospitalised or not). 

The prevalence of patients with preserved LVEF in our registry (30%) is similar to that reported by other authors [7] but less than that found in the Framingham and in the Minnesota studies [3, 12]. In the EuroHeart Failure Survey the proportion of patients with preserved LVEF (considered as LVEF>40%) was 46% [8]. In all those studies, patients were enrolled after an index hospitalisation and this may not reflect the real natural history of heart failure with preserved ejection fraction. The criterion for normal systolic function in our registry is LVEF ≥ 45% according to the majority of recent studies and as suggested by the current ESC Guidelines [19]. 

### Clinical Characteristics and Therapy

We found differences between groups in terms of age, sex, aetiology and co-morbidities. Patients with heart failure and preserved LVEF are older, they are more commonly women and more frequently the aetiology is of hypertensive origin. Other Spanish group have reported similar results [8]. Patients with preserved LVEF had a significantly higher rate of atrial fibrillation which may be a precipitant of clinical deterioration in this setting. 

Although there is currently no evidence available from randomised controlled trials on treatment of patients with preserved LVEF with ACE inhibitors or beta blockers, a considerable percentage of these patients were treated with such drugs. The percentage use of ACE inhibitors and beta blockers in patients with preserved LVEF was significantly lower than those with depressed LVEF, as clinical evidence is lacking but nevertheless still high, probably partly because of the ischaemic or hypertensive aetiology in many of these patients. These findings are consistent with those of the CHARM program in which the proportion of patients treated with diuretics, ACE inhibitors, spironolactone and digitalis decreased as LVEF increased [20]. In the recently published EuroHeart Failure Survey [8], the percentage of patients receiving ACE inhibitors or ARBs was 66% (81% in our registry) and the percentage of patients on beta blockers was 37% (66% in our study). The study reported herein shows that achieving adequate therapy, similar to that seen in clinical trials, is possible in a large number of centres and units with a very variable organization and structure. In fact, no significant differences were detected in the pharmacological treatment provided by the different participating centres, irrespective they are general or community hospitals. 

### Morbidity and Mortality

Our principal findings are related to morbidity and mortality. Patients with heart failure with preserved LVEF had complication rates that were similar to those of patients with reduced LVEF, including similar rates of death, admissions for worsening heart failure or for acute coronary syndrome.

Our study showed that patients with preserved LVEF had a high mortality rate during follow-up that were not significantly lower than those of patients with a reduced LVEF. Overall survival, admission due to worsening heart failure free survival and any cardiovascular event free survival were all similar between groups. 

Traditionally prognosis of patients with heart failure was related to the ejection fraction and mortality of patients with reduced LVEF was much higher than those with preserved LVEF [12, 15, 20-23], however, some other studies reported the opposite [7, 14]. More recently, in a substudy of the CHARM program [20] LVEF was an important predictor of mortality and ejection fraction was a poorer predictor of cardiovascular outcomes in those with an LVEF above 45%. Bhatia *et al. *[7] reported similar one-year mortality rates of patients presenting with new-onset heart failure with LVEF<40% and LVEF>50%. In the study by Senni *et al. *[3], survival at 6 years was not statistically different in heart failure patients regardless of the level of systolic function. On the contrary, Owan *et al. *[13] reported a slightly better survival among patients with preserved ejection fraction (EF>50%) in a retrospective analysis of patients hospitalized with decompensated heart failure. In the EuroHeart Failure Survey [8] mortality at 12-week follow-up was 10% in patients with preserved LVEF and 12% in patients with reduced LVEF which is very similar to the mortality rate of our study. Nonetheless, the characteristics of these patients differed to those of our patients, since patients in the EuroHeart Failure Survey were older (69 years old and 66 years old in our registry). Furthermore, patients in the EuroHeart Failure Survey were enrolled during hospitalisation due to heart failure whereas in our registry patients could be enrolled after admission to hospital due to heart failure or from the outpatient clinics. Thus, we cannot rule out the possibility of a selection bias due to the characteristics of our registry and the criteria for inclusion of the patients in the heart failure clinics, which resulted in patients having a lower risk.

Some authors have underlined the effect of coronary artery disease on the survival of patients with heart failure. The presence of coronary artery disease is an important prognostic factor; when patients with coronary artery disease are excluded, the annual mortality of patients with preserved LVEF is only 2%-3%. O’Connor *et al. *[24] observed that the severity of ischaemic disease was an independent risk factor with respect to the mortality of such patients, whether left ventricular systolic function was preserved or not. In addition, when these authors compared survival rates of patients with heart failure and reduced systolic function to that of patients with heart failure and preserved systolic function, the difference disappeared when adjustment (among other variables). In contrast, other studies report no differences in prognosis associated with ischaemic and non-ischaemic aetiologies [23, 25]. Setaro *et al. *[25] found that mortality at 7 years in patients with ischaemic and non-ischaemic aetiology was the same (46%). In our study, the ischaemic aetiology was an independent risk factor. 

Randomised studies which have compared the care provided by heart failure units with that afforded by the usual setting have shown a very significant reduction in admissions for worsening heart failure [26, 27]. Two randomised studies have recently been published which also showed a significant reduction in mortality, one of them a multicentre study in Spain [28, 29]. Thus, our results may be partly explained by such factor. However, the study by Atienza *et al. *[29] only included patients with heart failure who were discharged from the hospital and who therefore have a worse prognosis than patients in the BADAPIC registry, almost half of whom were outpatients with no recent admission. Despite these small differences between the two studies of randomised intervention and the observational BADAPIC study, the annual rates of mortality are between 5%-10% in all 3 studies, much lower than the annual mortality rates of 20%-30% in the previously mentioned population registries [2,8], and similar to those of clinical trials [15]. Thus, care of patients with heart failure in specialized clinics or units therefore seems to improve the prognosis of theses patients.

### Limitations

The main limitation of this registry, its observational and non-controlled character, introduces a possible selection bias when evaluating the results concerning the relative low rates of mortality and morbidity. The inclusion in our registry of both hospitalised and out-patients may affect the outcome as prognosis of out-patients is clearly better than those who have been admitted due to a decompensation. The results of randomised interventional studies with specific heart failure clinics support the idea that this type of care can improve the prognosis of patients with heart failure as educational and farmacological treatment may be improved. Our findings reinforce the need for new preventive strategies and new treatments specifically targeted at patients with congestive heart failure and preserved systolic function.

## Figures and Tables

**Fig. (1):**
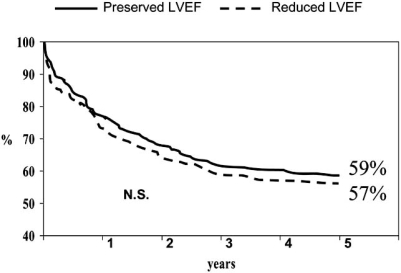
Kaplan-Meier unadjusted survival curves for patients with heart failure and preserved or reduced ejection fraction.

**Fig. (2):**
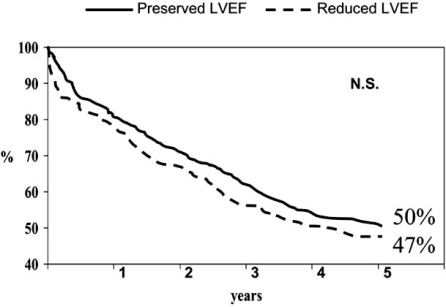
Kaplan-Meier unadjusted admission-free survival curves for patients with heart failure and preserved or reduced ejection fraction.

**Fig. (3):**
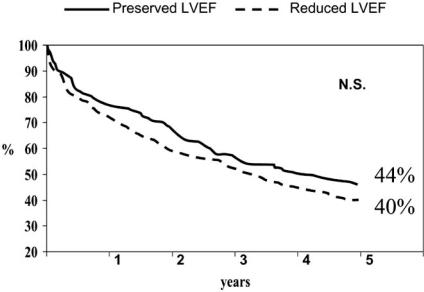
Kaplan-Meier unadjusted event-free survival curves for patients with heart failure and preserved or reduced ejection fraction.

**Table 1:** Clinical Features of 4720 Patients included in the BADAPIC Registry according to LVEF

	LVEF≥45% N=1416	LVEF<45% N=3304	p-value

Age, years (SD)	71(12)	64(12)	0.001

Sex Female (%)	53	28	0.001

Ejection Fraction, %(SD)	58(10)	30(7)	0.001

Aetiology (%)			
Ischaemic	25	44	
High blood pressure	31	10	0.001
Idiopathic Dilated cardiomyopathy	8	32	
Other	36	14	

High Blood Pressure (%)	66	49	0.001

Hyperlipidemia (%)	36	41	0.012

Diabetes Mellitus (%)	36	31	0.023

Coronary Artery Disease (%)	32	47	0.001

Prior AMI (%)	18	40	0.001

Coronary Revascularization	12	19	0.001

NYHA functional class (%)			
II	77	71	NS
III-IV	23	29	

Prior admission for heart failure (%)	68	72	NS

Cardiac rhythm on EKG (%)			
Sinus	54	70	0.001
Atrial Fibrillation	46	30	

Anemia (Hb<12 g/dl) (%)	23	17	0.05

Renal dysfunction (Cr>2mg/dl) (%)	9	10	NS

**Table 2:** Pharmacological Treatment following the Initial Visit, according to LVEF

	LVEF≥45% N=1416	LVEF<45% N=3304	p-value
Diuretics, n (%)	1218 (86)	2841 (86)	NS
Digoxin, n (%)	566 (40)	1619 (49)	NS
ACE inhibitors, n (%)	892 (63)	2709 (82)	0.001
ARB, n (%)	368 (26)	694 (21)	NS
Spironolactone, n (%)	1416 (30)	1487 (45)	0.001
Betablockers, n (%)	694 (49)	2445 (74)	0.001
Calcium antagonists, n (%)	354 (25)	264 (8)	0.05
Nitratates, n (%)	396 (28)	991 (30)	NS
Antiarrhythmics, n (%)	184 (13)	463 (14)%	NS
Anticoagulation, n (%)	651 (46)	1520 (46)	NS
Antiaggregants, n (%)	665 (47)	1586 (48)	NS
Statins, n (%)	396 (28)	1090 (33)	NS

**Table 3:** Incidence of Events for 100 Patients/Year of Observation in our Patients according to LVEF

	Total	Preserved Ejection Fraction	Reduced Ejection Fraction	Rates difference	p-value
Total Death	1416 (9.01)	440 (8.96)	976 (9.08)	0.12 (-0.34-0.67)	0.7894
Death due to CHF	912 (5.79)	281 (5.67)	631 (5.87)	0.20 (-0.40-0.79)	0.6756
Admissions due to CHF	1513 (9.61)	467 (9.4)	1046 (9.74)	0.31 (-0.39-0.95)	0,5687
Other CV admissions	418 (2.65)	129 (2.60)	289 (2.69)	0.09 (-0.58-1.12)	0.8765
Admissions due to ACS	312 (1.98)	96 (1.94)	216 (2.01)	0.07 (-0.71-1.21)	0.8863
Coronary revascularization	306 (1.96)	96 (1.94)	210 (1.96)	0.02 (-0.60-1.01)	0.9012
Heart Transplant	52 (0.33)	7 (0.15)	45 (0.42)	0.27 (-1.01-1.78)	0.2346
ICD implant	112 (0.71)	5 (0.10)	107 (0.99)	0.89 (-1.11-2.23)	0.078
Any event	1880 (11.94)	582 (11.74)	1298 (12.08)	0.34 (-0.37-1.01)	0.5853

**Table 4:** Univariate Cox Regression Analysis

Characteristics	Deads (n=1416)	Alive (n=3304)	p-value
Age, years (SD)	71(12)	64(10)	0.001
Gender, Male (%)	66	65	0.44
Hypertension(%)	60	54	0.05
Hypercholesterolemia (%)	53	39	0.001
Diabetes Mellitus (%)	36	30	0.003
Prior AMI (%)	42	30	0.001
Prior revascularization (%)	19	15	0.002
Prior CHF admissio (%)	69	70	0.34
NYHA Class III-IV (%)	27	22	0.05
Ischemic aetiology (%)	48	39	0.001
Atrial Fibrillation (%)	35	30	0.04
EF, %(SD)	33(14)	38(13)	0.001
EF<45% (%)	80	72	0.003
Hb<12 gr/dl (%)	24	15	0.001
Cr>2 mg/dl (%)	13	7	0.003
Diuretics (%)	85	86	0.23
Digoxin (%)	45	47	0.51
ACE inhibitors (%)	74	78	0.06
ARB-II (%)	21	22	0.56
Spironolactone (%)	40	36	0.04
Betablockers (%)	60	73	0.001
Calcium channel blockers (%)	13	14	0.61
Antiplatelets (%)	50	42	0.002
Nitrates (%)	34	27	0.002
Statins (%)	22	37	0.001

**Table 5:** Adjusted Hazard Ratios for Predictors of Mortality of Patients with Congestive Heart Failure from Multivariate Cox Regression Analysis

Variable	HR	95% CI	p-value
Age	1.68	1.18-2.05	0.0001
Ischemic aetiology	1.09	1.04-1.95	0.008
Anemia	1.62	1.23-2.01	0.0001
Creatinine>2 mg/dl	1.41	1.10- 2.57	0.001
Betablockers	0.81	0.48-0.95	0.003
Statins	0.75	0.45-0.89	0.002

## APPENDIX

### Participating Centres and Investigators of the BADAPIC Registry

1. Hospital General de Albacete: Pablo Domínguez Barrio.
2. Fundación Hospital Alcorcón: Elena España Barrio, Elena Batlle López.
3. Hospital General de Alicante: Francisco Sogorb Garri, Vicente Climent Payá.
4. Hospital de Antequera: Jesús Alvarez Rubiera, Alvaro Rubio Alcaide.
5. Hospital San Agustín de Avilés: Gerardo Casares García.
6. Hospital Infanta Cristina de Badajoz: León Martínez de la Concha.
7. Hospital Can Ruti de Badalona: José Lupón Roses, Teresa Pajarón Rodríguez.
8. Hospital San Eloy de Baracaldo: Javier Andrés Novales.
9. Hospital Vall d´ Hebrón de Barcelona: Stella Méndez, Enrique Galve.
10. Hospital de Terrassa: MA de Miguel, David López Gómez.
11. Hospital Mutua de Terrassa: Leandro Saenz, Amparo Alvarez.
12. Hospital Sant Pau de Barcelona: Domingo Ruiz Hidalgo, Josep Antón Montiel Dacosta.
13. Hospital Clinic i Provincial de Barcelona: Eulalia Roig Minguell, Alfredo Cupoletti Beange.
14. Hospital Sacrat Cor de Barcelona: Francesc Rossell Abaurrea, Cesar Morcillo Serra.
15. Hospital de Basurto de Bilbao: Nekane Murga Eizagaechaverria, Inmaculada Lluis Serret.
16. Hospital San Pedro de Alcántara de Cáceres: Concepción de la Concepción Palomino, Yolanda Porras Ramos.
17. Hospital General de Castellón: José Luis Diago Torrent, Alex Navarro Bellver.
18. Hospital Reina Sofía de Córdoba: Manuel Anguita Sánchez, Soledad Ojeda Pineda.
19. Hospital de Elche: Fernando García de Burgos y de Rico, Alejandro Jordá Torrent.
20. Hospital de Galdakao: Javier Zumalde Otegui, Alberto Salcedo Arruti.
21. Hospital de Gandía: Plácido Orosa Fernández, Catherine Lauwers Nelisen.
22. Hospital Virgen de las Nieves, Granada: Oscar Baun, José Luis Ventin Pereira.
23. Hospital General de Granollers, Barcelona: Santiago Montull Morer, Rosa Guitard.
24. Hospital del SAS de Jerez: José Carlos Vargas Machuca, Fernando García-Arboleya Puerto.
25. Hospital de Bellvitge: Nicolás Manito Lorite, Edgardo Kaplinsky.
26. Complejo Hospitalario de León: Julián Bayon Fernández, Manuela Montes Montes.
27. Hospital La Paz, Madrid: Isidoro González Maqueda, Gabriela Guzmán Martín, Llanos Soler Rangel, Francisco Arnalich Fernández.
28. Hospital Severo Ochoa, Leganés: Ana Isabel Huelmos Rodrigo, Angel Grande Ruiz.
29. Hospital de la Princesa, Madrid: Mercedes Fernández Escribano.
30. Hospital Costa del Sol, Marbella: Emilio González Cocina, Francisco Torres Calvo.
31. Hospital Carlos Haya, Málaga: Manuel de Mora Martín, José María Pérez Ruiz.
32. Hospital Virgen de la Victoria, Málaga: Eduardo de Teresa Galván, Encarnación Molero Campos, Manuel Jiménez Navarro.
33. Hospital Comarcal de Mendaro: Esther Recalde del Vigo, Nicolás Gurrutxaga Arrillaga.
34. Hospital Provincial Santa María Madre, Orense: Miguel A. Pérez de Juan, Manuel de Toro Santos.
35. Hospital Central de Asturias: Beatriz Díaz Molina, José Luis Rodríguez Lambert.
36. Hospital Río Carrión, Palencia: Fausto Librada Escribano.
37. Hospital General de Mallorca: Josefina Gutiérrez Alemany.
38. Hospital de Santa Bárbara, Puertollano: José Portillo Sánchez .
39. Hospital Sant Joan de Reus, Tarragona: Francesc Marimón Cortés, Oscar Palazón Molina.
40. Hospital Clinico Universitario de Salamanca: Pedro Luis Sánchez Fernández, Francisco Martín Herrero.
41. Hospital Donosita de San Sebastián: Ramón Querejeta Iraola, Eloy Sánchez Haya.
42. Hospital Marqués de Valdecilla, Santander: José Ramón Berrazueta Fernández.
43. Hospital Clínico Universitario de Santiago de Compostela: José R. González Juanatey, Inés Gómez Otero.
44. Hospital Universitario de Valme, Sevilla: Juan C. Beltrán Rodríguez, Luis Pastor Torres.
45. Hospital Virgen del Rocío, Sevilla: Angel Martínez Martínez.
46. Hospital Joan XXIII de Tarragona: Alfredo Bardají Ruiz, Ramón de Castro Aritmediz.
47. Hospital Sant Pau i Santa Tecla, Tarragona: Lluis Carles Olivan Sayrol, Juan Carlos Soriano Giménez.
48. Hospital Universitario de Canarias: Antonio Lara Padrón, Francisco Marrero Rodríguez.
49. Hospital General de Valencia: José Antonio Velasco Rami, Francisco Ridocci Soriano.
50. Hospital La Fe, Valencia: Luis Almenar, Joaquín Rueda Soriano.
51. Hospital Doctor Peset, Valencia: Begoña Sevilla Toral, Antonio Salvador Sanz.
52. Hospital Clinico Universitario de Valladolid: Luis de la Fuente Galán.
53. Hospital Mexoeiro de Vigo: Francisco Calvo Iglesias, José Luis Escribano Arias.
54. Hospital de Txagorritxu, Vitoria: Fernando Arós Borau.
55. Hospital Clinico Universitario Lozano Blesa, Zaragoza: Alfonso del Río Lligorit, Antonio San Pedro Feliú.
56. Hospital Miguel Servet, Zaragoza: Marisa Sanz Julve, Teresa Blasco Peiró.
